# A Case

**Published:** 1855-09

**Authors:** G. W. Parkins

**Affiliations:** Illinois


ARTICLE II.—*A Case.* By G. W. Parkins, M. D, of Illinois.

About three years since, Mrs. B———, a very respectable lady of Tremont, Tazewell county, Ill., had a severe attack of dysentery, from and after which her catamenia ceased entirely, and so continued for two years, when she was married to Mr. B. At this time her menses returned for two or three times only, then disappeared again. During these two years, there took place a gradual hypogastric enlargement, steadily increasing, but painless, occasioned by what appeared to be a hard tumor in that region. After marriage this tumor grew more rapidly, filling up the umbilical region, and encroaching on the epigastrium, and, as she was affected by none of the ordinary signs of pregnancy, except the abdominal enlargement and the cessation of the menses, both of which existed before marriage, her physician, Dr. Shaw, suspected ovarian dropsy, or uterine disease. He advised her to visit St. Louis for medical advice, and about the middle of June last, Mr. B. accompanied her thither, where she was examined by Dr. Pope, who made an exploratory puncture into the tumor. He decided that it was a fleshy or fibrous development within the womb —that pregnancy might co-exist, but it was not probable, and that there was little or no ground to justify a hope that an operation would be beneficial. In a desponding mood, they now took boat for home. During the first day she had occasional pains in the hypogastrium, which increased in frequency, and severity, till the afternoon of the second day, when they had become so severe that she determined to land at our place, Havana, believing the cause of her sufferings to be the jarring motion of the boat. This was July 2d. One hour after landing, I was called in, and found Mrs. B. far advanced in labor, but *she* did not think she was pregnant at all. She was 39 years old, and this her first confinement, which I thought accounted for the severe and protracted labor. In two hours Mrs. B. gave birth to a fœtus which appeared to have arrived at full term, but in which life was extinct, the bones of the cranium being much distorted.

But there still existed great abdominal prominence, so much so that my first impression was, that there was another child in utero.

I called in Dr. John McCowen, and after a careful examination, we concluded the extraordinary prominence was owing to the existence of a tumor about the size of an adult head The most prominent part of the tumor, that wherein the puncture had been made, was tender on touch or pressure, otherwise the patient was easy. But during the four days which she survived parturition, there were no uterine contractions, resembling labor pains; no secretion of milk; pulse 80 per minute and weak, and increasing in number, and weakness, till death closed the scene; the countenance cadaverous; the surface bathed in cold perspiration; hiccough was a prominent and distressing symptom; and finally death supervened, on the 6th of July, under all the characteristic symptoms of mortification of some portion of the abdominal vicera.

Dr. Nastich saw the patient on the 4th, and said there was a second dead child in the womb, which should be removed without delay, to which Mrs. B. did not consent. Therefore we thought best, in self-defence, to institute an autopsy. On laying open the abdominal cavity, the womb was found very much enlarged, occupying the hypogastric and umbilical regions almost completely. In external appearance, it was firm, bilobed, one lobe occupying the fundus, the other, the inferior and anterior portion of the uterus; and in color it was nearly natural, except the interior part of the inferior lobe, which rested down against the os pubis. This was the part punctured, and around the puncture mortific ition had taken place. The edges of the wound, having taken on malignant action, were putrid, black, very much thickened and everted. The surface was livid and black for the space of four inches in diameter.

From the fundus uteri, there grew or pushed up into the cavity of the abdomen, three tumors about the size of goose eggs, having the peritoneal envelop, common to them and the womb. They were solid, somewhat elastic, and of similar internal construction to the tumor, to which they were attached. The uterus was no where less than one inch thick, and the two principal lobes were four to five, each of which was a fleshy, fibrous mass, of a whitish-yellow, and sometimes of a reddish-yellow appearance, with here and there a small cyst containing a spoonful or less of serum. The mucous membrane of the womb looked healthy, the uterine cavity was long but much contracted—the placenta was not yet attached. was about 7 or 8 inches long, and very slender, $\frac{1}{2}$ to $\frac{3}{4}$ of an inch thick, and from 1 to 2 inches wide; it was lying loose in the cavity of the womb, which latter did not seem capable, owing to its enormously thickened condition, of any further contraction, by which alone the placenta could be detached and thrown off.

Some adhesions existed between the posterior surface of the womb, and the large intestines—otherwise the abdominal viscera were perfectly healthy. The uterus weighed 9$\frac{3}{4}$ lbs.

As the entire womb was affected more or less, and as it had many of the internal and external characters of health, the diseased developement was probably the result of perverted action of the uterine lymphatics.

It did not appear that death was in any way connected with the retention of the placenta

The patient might have lived longer, possibly some years, had she not become pregnant, and she *might* not have died, neither been confined, quite so early, had the tumor not been punctured; though in any event life could not have been protracted long.

*Cure of Diabetes Mellitus.*—By Dr. Zipfehli.—The diabetes here mentioned was cured in the short space of three months and a few days by the use of cod-liver oil in full doses, and conjoined with a diet of animal food.—(*Gaz. Med.*)

*Observation.*—"A journalist, 25 years old, entered the hospital to be treated for the itch. It was discovered that he had diabetes since the previous September. Two or three spoonsful of cod-liver oil, a day, were first prescribed, to be increased *ad libitum.* The patient grew so fond of the remedy that he consumed about a half pound of it in two days. On the 30th of May, 1854, he was able to leave the hospital, entirely cured; he had regained his flesh and there was no trace of sugar in his urine; in all, he took thirteen pounds (*litres*) of the oil. It is possible that the rapidity of the cure in this case was owing to the fact that the diabetes was recent. On the other hand, the patient was a very poor man, who lived in the midst of privations and was a brandy drinker; the good diet upon which he was plac d in the hospital. without doubt contributed greatly to his recovery.—*Gaz. Med.*

